# A qualitative, participatory study to identify barriers and facilitators to women’s uptake of National Health Hotline Solutions in Malawi and Mozambique

**DOI:** 10.1093/oodh/oqaf035

**Published:** 2026-02-06

**Authors:** Jocelyn Powelson, Lucky Gondwe, Eliud Akama, Hannah Kachule, Katie Nkhonjera, Lidia Jahar, Jessica Mayenda, Edwin Mulwa

**Affiliations:** Research, Evidence & Learning Team, VillageReach, 210 S Hudson St Suite 307, Seattle, WA 98134, United States; Independent Consultant, VillageReach, PO Box 31348, Lilongwe 3, Malawi; Department of Global Health, University of Washington School of Public Health, 3980 15th Ave NE, Seattle, WA 98195, United States; Independent Consultant, VillageReach, PO Box 31348, Lilongwe 3, Malawi; Independent Consultant, VillageReach, PO Box 31348, Lilongwe 3, Malawi; Independent Consultant, VillageReach, Rua Beijo da Mulata nº 258, Somerschield II, Maputo, Mozambique; Digital Solutions, VillageReach, 11th Floor (West Wing), Riverside Square, 104 Riverside Drive, Nairobi 00100, Kenya; Digital Solutions, VillageReach, 11th Floor (West Wing), Riverside Square, 104 Riverside Drive, Nairobi 00100, Kenya

**Keywords:** telehealth, qualitative research, gender, participatory research, mHealth, health hotline

## Abstract

Gender disparities in digital health service utilization remain significant, particularly in low-resource settings. In 2024 in Malawi and Mozambique, women constituted only 30% and 10% of callers, respectively, to national health hotlines, which provide health information via hotline agents and Interactive Voice Response (IVR) messages. This study explored factors influencing women’s uptake of health hotline services in Malawi (Lilongwe and Mzimba North districts) and Mozambique (Quelimane) using a community-based participatory approach. Methods included key informant interviews with hotline agents and Ministry of Health stakeholders and in-depth interviews and voice diaries with women in the study sites. Jhpiego’s Gender Analysis Framework guided tool development and coding, and data were analyzed using ATLAS.ti v23. Findings were mapped to a user journey based on UNICEF’s Journey to Health and Immunization framework. Key barriers included low awareness of services, lack of access to personal phones, household responsibilities, socio-cultural preferences for in-person care, infrastructure limitations, and call center staffing shortages. Unique barriers in Mozambique included limited language options and narrow IVR message topics. Facilitators included strong social networks enabling phone access, trust in government services, the perceived value of health information, 24/7 service availability, and positive interactions with hotline agents. While focused on the health hotline implementations in Malawi and Mozambique, these findings are broadly applicable to enhancing women’s engagement with digital health solutions in similar contexts.

## INTRODUCTION

Barriers to equitable access to quality, in-person health services persist, especially in low- and middle-income countries (LMICs). With the technological advancements of the past two decades, there is increasing recognition of the potential for digital and mobile health solutions to extend the reach of the health system, improve efficiency and cost-effectiveness of health services, increase health workforce capacity, and improve quality of services [1]. mHealth interventions have also been shown to positively influence male and female cooperation and communication around health seeking behaviors [2]. Despite the potential of digital and mobile health solutions, their uptake remains low for some populations. Barriers to uptake include low digital literacy, language barriers, limited access to mobile phones or the internet, concerns about data privacy, poor tailoring of solutions to meet the needs and preferences of target users, and insufficient technical capacity [3–6]. These challenges are exacerbated by socioeconomic factors that disadvantage low income and rural communities [7].

Gender disparities in the adoption of digital health tools are especially pronounced due to unique obstacles that many women in LMICs face. Barriers that impede women from accessing and utilizing mobile health solutions include lower mobile phone ownership, lower education levels, lower literacy levels and language skills, lack of awareness of mobile health solutions, limited autonomy of women in decision-making about technology use or finances, cultural norms that restrict women from freely engaging with communication platforms, failure to acknowledge and design solutions to accommodate a heterogenous population of women users, and greater time burdens due to caregiving responsibilities and other work [4, 8–11]. Addressing these barriers is critical to ensuring that digital health solutions achieve their intended equity and public health goals.

Malawi and Mozambique are two of the many countries in sub-Saharan Africa where health hotlines have been implemented to extend the reach of health services and information. Malawi’s Chipatala Cha Pa Foni (Chichewa for ‘Health Center By Phone’, CCPF), established in 2011, is a national health hotline that offers health information to users in multiple languages and on a variety of topics through a 24/7 hotline staffed by trained agents as well as through pre-recorded informational messages accessed through an Interactive Voice Response (IVR) system. CCPF services are free for subscribers of Airtel, one of two major telecom providers in Malawi. In 2022, an estimated 70% of men and 48% of women in Malawi owned a phone [12].

Mozambique’s AlôVida hotline, which began operations in 2002, provides similar services to users in Mozambique. While the service offerings in Mozambique have since expanded, at the time of data collection, users could access health information in multiple languages and on a variety of topics through a 24/7 hotline or could access informational IVR messages in Portuguese on immunization and COVID. AlôVida services are free for subscribers of all telecom providers in Mozambique. In 2022, an estimated 72% of men and 59% of women in Mozambique owned a phone [13]. While both hotlines aim to reach all citizens with health information, demographic data from hotline callers in 2024 showed significant gender gaps in both countries; women accounted for only 30% and 10% of callers for whom gender data was collected in Malawi and Mozambique, respectively. The objective of this study was to identify the most common and significant barriers affecting women’s uptake of the service. An exhaustive list of these would have been desirable, but this is among the limitations of this study. This approach aligned with the applied purpose of the research: to inform improvements to the national health hotlines.

## MATERIALS AND METHODS

### Study design

We conducted a qualitative study using participatory research methods to generate actionable insights grounded in the lived experiences of health hotline users and service providers. Participatory approaches engage community representatives throughout the research process to generate contextualized and community-centered knowledge [14, 15]. They prioritize methods that empower participants to express themselves in their own words [15, 16].

We recruited one woman in Mozambique and two in Malawi as community researchers. The women had all previously used the hotline services and lived in or near the study sites. They were trained in research ethics and qualitative methods, contributed to tool development and recruitment, led data collection, and participated in analysis. The researchers leveraged their local insights and experiences throughout the research process.

**Figure 1:**
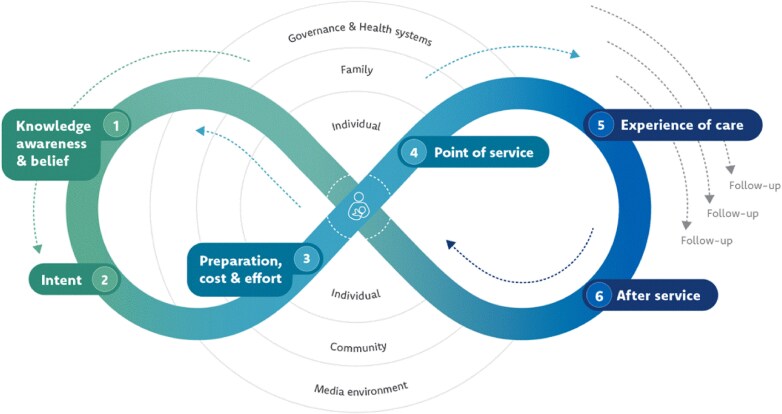
UNICEF Journey to Health & Immunization framework. UNICEF, Demand for health services field guide. New York: UNICEF, 2018.

Data collection and analysis were guided by Jhpiego’s Gender Analysis Framework [17], which explores gender norms and contextual factors across the four domains of **access to assets**, **beliefs and perceptions**, **practices and participation**, and **institutions, laws and policies**. Within each domain, we identified key demand-side and service-side facilitators and barriers to women’s uptake of the hotlines. Further, to make the findings more actionable for interventions targeting different stages of the user journey, we mapped the insights onto a modified version of UNICEF’s ‘Journey to Health & Immunization’ framework (Fig. 1) which we adapted to reflect digital health experiences rather than immunization [18].

### Study setting, population, and recruitment

Data were collected between August 2023 and May 2024 in Mozambique and between September 2023 and February 2024 in Malawi. Participants included key informants in the capital cities where the call centers are based and women from both rural and urban communities. In Mozambique, women were recruited from communities in Namacurra District and Quelimane City in Zambézia Province. In Malawi, women were recruited from communities in Mzimba North and Lilongwe Districts. Sites were purposively selected in coordination with national and sub-national Ministry of Health (MoH) stakeholders.

Key informants included call center agents and other MoH stakeholders involved in the management and operations of the national health hotlines. To be eligible, agents needed at least six months of experience at the call center, and other MoH stakeholders needed a scope of work that relates to operations or management of the health hotlines. We purposively recruited a mix of male and female hotline agents who had significant experience with the hotline as well as the MoH stakeholders who played the biggest roles in overseeing management of the hotline.

Eight key informants in Malawi and seven in Mozambique participated (Table 1). Three hotline attendants (two female and one male) and one hotline supervisor in each country participated along with other stakeholders from the relevant MoH departments involved in management and promotion of the hotlines. Hotline attendants in Malawi had worked at the call center for 2–5 years, while those in Mozambique had worked at the call center for 4–22 years.

**Table 1:** Characteristics of key informants.

	Malawi (n = 8) N (%) or median (IQR)	Mozambique (n = 7) N (%) or median (IQR)
Role Hotline attendant Hotline supervisor Other stakeholder	3 (38) 1 (13) 4 (50)	3 (43) 1 (14) 3 (43)
Total years of experience for hotline attendants	2 (2–5)	19 (4–22)
Sex: male Hotline attendant Hotline supervisor Other stakeholder	1 (33) 1 (100) 1 (25)	1 (33) 1 (100) 3 (100)

The eligible study population for women participants included all women in the communities, regardless of phone ownership or prior hotline use. The community researchers used a purposive sampling approach to recruit and enroll women from a variety of age ranges. Community health volunteers from the study communities assisted the researchers in recruiting eligible women from different age ranges, as they knew which women were likely available to participate and where they were located.

Thirty women participated in the study, including 20 in Malawi and 10 in Mozambique, with equal rural/urban representation (Table 2). Women in Malawi ranged from 22 to 61 years of age, and those in Mozambique ranged from 20 to 42 years of age. All participants cared for at least one child. Women represented a diverse set of other characteristics, including marital status, education level, employment, and mobile phone ownership.

**Table 2:** Characteristics of women participants.

	Malawi (n = 20) N (%) or median (IQR)	Mozambique (n = 10) N (%) or median (IQR)
Geography Rural Urban	10 (50) 10 (50)	5 (50) 5 (50)
Age	30.5 (28.5–38.3)	27.5 (23–30)
Marital status Married Unmarried	17 (85) 3 (15)	4 (40) 6 (60)
Number of children in care	3 (2–4.5)	2 (1–5)
Highest level of education Primary Secondary Bachelor	11 (55) 9 (45) 0 (0)	4 (40) 4 (40) 2 (20)
Income-generating job Farming Business Other None	11 (55) 4 (20) 2 (10) 3 (15)	1 (10) 3 (30) 1 (10) 5 (50)
Personal phone ownership Smart phone Basic phone None	4 (20) 12 (60) 4 (20)	1 (10) 6 (60) 3 (30)

### Data collection

Community researchers collected demographic information and obtained written informed consent prior to interviews. Tools were piloted and refined prior to data collection. Interviews were conducted in Portuguese in Mozambique and in Chichewa, Tumbuka, or English in Malawi, depending on participant preference.

#### Key informant interviews

Semi-structured interviews with key informants explored hotline design and aims, promotion and demand generation strategies, agent training, data use, experiences of interactions with female versus male callers (for hotline agents), and perceived barriers and facilitators to women’s uptake of services.

#### In-depth interviews

Semi-structured in-depth interviews with women participants were structured around the Gender Analysis Framework and focused on how they access health information and services, phone and resource access, perceptions of health information sources, and their experiences or expectations relating to accessing health information through a hotline.

#### Voice diaries

We employed a participatory narrative method [16] that we termed ‘voice diaries’ to allow women participants from a range of literacy levels to share their stories of their hotline experiences. Following the in-depth interviews (IDI), the researchers guided participants on how to call the hotlines and provided simple voice recorders and pictorial instructions. Participants were asked to call the hotline at least three times over the following week and record a ‘voice diary’ describing each experience. They were prompted to describe what kind of services or information they tried to get, their level of satisfaction, and likes and dislikes about the experience. Women in Malawi recorded an average of 2.2 voice diaries each (range 1–4) while those in Mozambique recorded an average of 9 voice diaries each (range 3–23).

#### Follow-up debrief interviews

The researchers returned after one to two weeks to collect the voice recorders and conduct a shorter semi-structured debrief interview. These debriefs largely focused on the ‘Institutions, Laws and Policies’ domain of the framework, exploring how the hotlines’ structures, processes, and systems shaped user experiences. Participants also shared recommendations for improvement.

All women respondents participated in all three methods, including IDIs, voice diaries, and follow-up debrief interviews. JP reviewed the first set of transcripts from each researcher and provided feedback to improve the quality of subsequent interviews. Throughout the data collection period, the research team debriefed on the incoming data and discussed areas for further probing. Data collection continued until we observed recurring themes and saturation of key issues. After interviews with 30 women and 15 key informants, common patterns and barriers were consistently emerging.

### Data analysis

Interviews and voice diaries conducted in English and Portuguese were transcribed and translated using the Whisper speech recognition model, and transcriptions were reviewed for accuracy. Those conducted in Tumbuka and Chichewa were transcribed and translated by the community researchers.

A codebook was developed deductively using the Gender Analysis Framework and then expanded inductively following review of initial transcripts (Supplemental Material Appendix A). JP, EA, and LG coded two transcripts together, then individually coded three additional transcripts in ATLAS.ti V.23 and reviewed and discussed the coded transcripts to align on coding styles and interpretations. They then divided and independently coded the remaining transcripts. Documents were grouped by participant type (key informants, rural women, and urban women) and country.

We conducted a thematic analysis of the data [19]. ATLAS.ti’s AI analysis tools generated preliminary summaries for each code by document group. EA then reviewed each code summary against the raw data and edited or added additional insights and representative quotations. Then JP, EA, LG, HK, and KN independently reviewed the code summaries and identified key findings, facilitators, and barriers for each domain of the framework and each participant group. They then met to discuss key findings and used a Miro board to visualize connections between findings across the framework domains to generate themes. JP, EA, and LG then mapped the findings onto the adapted UNICEF Journey to Health framework to better capture how the barriers and drivers of uptake interact across the different stages of the user journey.

### Ethics approval

This study involved human participants and was approved by the Institutional Review Board of the Malawi National Health Sciences Research Committee (protocol code 23/04/4066 approved on 04 August 2023) and by the Institutional Review Board of the Mozambique National Bioethics Committee for Health (Ref: 369/CNBS/23 approved on 10 July 2023). Participants gave written informed consent (signed or thumbprint) to participate in the study before taking part.

## RESULTS

**Figure 2:**
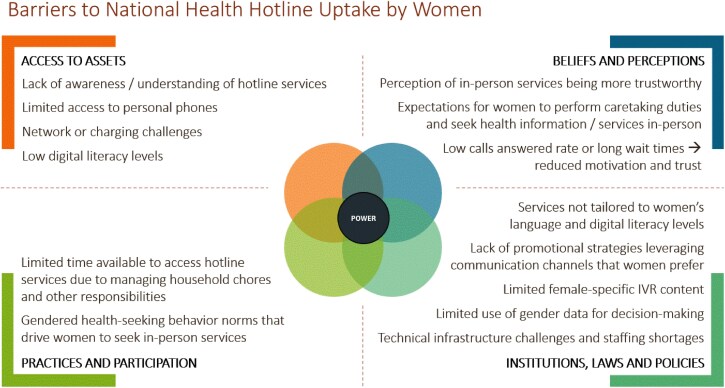
Barriers to uptake of National Health Hotlines by women.

**Figure 3:**
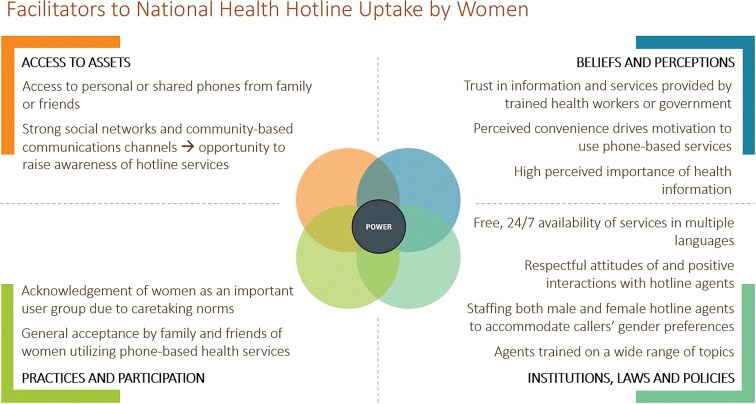
Facilitators to uptake of National Health Hotlines by women.

Barriers and facilitators to women’s uptake of national health hotline services emerged across the categories of the Jhpiego Gender Analysis Framework (Figs. 2 and 3). These facilitators and barriers are described in more detail in the themes below, organized by the stages of the UNICEF Journey to Health framework. Insights shared by key informants and women participants largely aligned. Women contributed additional details around how health hotlines fit within their daily routines, social norms, and health behaviors, and key informants provided more explanation of supply-side challenges that impact user experiences. Unless otherwise indicated, the findings apply to both Malawi and Mozambique. Pseudonyms have been used to protect participant confidentiality.

### Knowledge, awareness, and belief

*Key Finding: Health hotline services were not adequately promoted through women’s preferred communication channels, leaving women unaware of the service offerings.*

Awareness of the hotline services was low, particularly in Malawi. While many women respondents in Mozambique had heard of AlôVida, few understand what services it offered or that it was free and therefore had never tried to call the hotline. This barrier alone likely accounted for a large portion of the gender gap in hotline usership.

‘No [I haven’t heard about CCPF]. I feel like there has not been any sensitization here in our community, and this is a new initiative for me. It’s my first time hearing about it but maybe if there was some sensitization then it would have really helped to spread [awareness].’ – Chikondi, Malawi

‘I've only heard of this service, about AlôVida. But what they provide, I don't know.’ – Isabel, Mozambique

Women participants reported typically relying on health workers, community leaders, friends, and radio for health information—sources they trust and with whom they have personal connections. Participants perceived that current promotional strategies, which focus on TV and radio, reach men more than women. They recommended using word-of-mouth, community meetings, church gatherings, and facility-based health talks and posters to better reach women.

*Key Finding: Perceptions of trust and privacy varied across users and contexts, shaping women’s engagement with the national health hotlines in different ways.*

Women respondents’ trust and confidence in the services and information provided by the hotlines were often bolstered by their association with the government and by the health training of hotline agents. In Malawi in particular, women described the government affiliation as reassuring and a sign of credibility.

‘I would trust the information because as you mentioned this is a service that works in the whole of Malawi, and I trust that the Ministry of Health is involved in this service so I would trust the information.’ – Fanny, Malawi

Most felt comfortable sharing personal information and found the advice credible, citing agents’ health expertise and confidentiality. However, some women preferred in-person interactions with health workers, which felt more familiar and reassuring. When asked if she would feel comfortable sharing her health information with an AlôVida agent, a respondent replied, ‘No… If I want to share something intimate, I need to have confidence. Sometimes we don’t even know who we are talking to [on the hotline]. Not everyone keeps a secret, right?’

### Intent

*Key Finding: Cultural and gender norms placed family health responsibilities on women but limited their power to make decisions or access the resources needed to seek information and services.*

Participants reflected that social norms placed caretaking burden on women but did not empower them with the resources or decision-making power to carry out those responsibilities. Some faced resource barriers, such as not owning a phone, which restricted their ability to use the hotline services independently. Others described that decision-making about spending typically rests with men, limiting women’s ability to pay for transport to access facilities or services. This made hotlines appealing for them as a lower-cost, convenient option for health information, though dependent on phone access.

‘One thing I liked about [CCPF] is that it could be really beneficial to us women since we care mostly for our family’s health rather than men. We feel like it is our responsibility so it could really help us save the time we could spend travelling to the health facility to get help and just use the hotline to get help.’ – Esther, Malawi.

*Key Finding: Women used health hotlines to conveniently manage caregiving responsibilities without needing to visit facilities, while respondents reported that men are motivated by perceived privacy of hotlines and general discomfort with in-person services.*

Call center agents reported that women use health hotlines to access timely, reliable health information without the burden of traveling to a facility, especially when juggling household duties or facing resource constraints. The hotlines were perceived as a tool for women to support informed decision-making and address general health concerns promptly.

‘Whatever the symptom, even if it is insignificant, women rush to the health center. But if they had this knowledge that there is a line that I can call to find out if it is imperative for me to go to health center… this can very well reduce the demand in health care units.’ – Key informant, Department of Health Promotion, Mozambique MoH.

In contrast, key informants reported that men particularly valued the privacy and discretion offered by the hotline, which allowed them to seek information without social judgment. While the lack of a relationship with the hotline agents was a source of mistrust for some women respondents, the same anonymity was perceived as an advantage for male users.

‘The man can easily be in his private place, call the line, get clarifications there and decide that he does not need to go to the health unit... Because our health care facilities are not often humanized for men.’ – Key informant, Department of Health Promotion, Mozambique MoH.

They felt that men typically delay visiting health facilities for as long as possible. As a result, the hotline agents reported that women tend to call about preventive health topics, while men more often seek advice on acute health issues.

‘I would say men are not seeking advice as such, but they call because they have a problem, while women call because they just want guidance or something or just information because they don’t have it.’ – Hotline agent, Malawi.

*Key Finding: Stakeholders held inconsistent views on target populations for health hotline services, potentially impacting their motivation and willingness to make structural changes to address gender barriers**.***

Both countries’ strategic documents state that the telehealth services are intended to reach all citizens with free health information. Further, one of the priorities listed in the AlôVida 2022–26 Strategic Plan is to ‘increase the coverage of AlôVida services, particularly with strategies that allow for greater gender inclusion.’ While some informants felt it important to address gender disparities amongst users, others expressed satisfaction with high male engagement since they felt that men are generally underreached by primary health care services due to the gendered differences in health-seeking behavior norms.

‘In terms of [national population] proportion, we have almost the same percentage. There are a little more women than men in our country. This already shows that there is fragility here. We need to do additional work so that women can utilize our services.’ – Key informant, Health Promotion Department, Mozambique MoH.

‘[AlôVida] is trying to reach mainly men, from teenagers, young people, men, because we know that this class or this gender has some difficulty accessing our health services. So for them it is much easier, in the comfort of their home, with their privacy guaranteed, to get information about their health.’ - Hotline agent, Mozambique.

Key stakeholders involved in the hotlines held differing perspectives on target populations, some of which did not align with the hotlines’ strategic objectives. This lack of alignment may have limited some stakeholders’ motivation to address gender-specific barriers or use gender-disaggregated data for service improvements. Informants also reported resource constraints which hinder structural changes needed to make hotlines more accessible to women.

### Preparation, cost and effort

*Key Finding: Limited access to phones, charging, network availability, and SIM cards hindered some women from accessing hotline services, but social support networks helped to overcome these challenges.*

Many of the key informants cited gendered differences in phone ownership as a barrier to women’s utilization of services.

‘If there’s one phone in a home, it would be with a man rather than with a woman. So, access to the phone might have a bearing on why men are using it more than women. If I have a question, I’ll [call and] ask. But my wife will have to wait for me to come home. Or, we have to be together for her to access the information.’ – Key informant, Directorate of Curative and Medical Rehabilitation Services, Malawi MoH.

Women who had their own phones, especially those in the rural sites, experienced other challenges such as lack of electricity for charging and poor network coverage. CCPF services were further limited for users without AirTel SIM cards.

Social support networks helped bridge resource gaps; the women participants who did not have their own phones reported that they usually had access to shared phones from family members or friends, though not necessarily when or where they needed them.

‘When we want to talk to the health providers, we borrow a phone and return it after we use it… The time [we have access to it] is limited because the phone belongs to another person, so the owner takes it when they are going out. So, when there is an urgent matter to attend to, we go to borrow the phone.’ – Ruth, Malawi.

Hotline agents also reported men calling the hotline on behalf of their wives or other female family members, reflecting both practical constraints and gender norms around phone ownership and information-seeking.

*Key Finding: Competing priorities resulted in women having limited time to access health services; this served as a potential motivator due to the convenience of not needing to visit a facility but was also a demotivator when the user was experiencing long wait times or repeated disconnections.*

Most women respondents reported having heavy workloads including household chores, childcare, farming, and business, which limited their time to seek health information and services. This made hotlines attractive for the participants, as they could save travel time or access information outside of health facility hours.

‘I am ready to use [CCPF] because it seems like it would be very helpful and useful. Sometimes I may face a challenge in the middle of the night, and I can’t travel to the hospital so this phone will help to access help from health personnel, so it is welcoming for me.’ – Thoko, Malawi.

Despite the potential for convenience, long wait times, repeated disconnections, and unanswered calls left some women participants feeling that using the hotline service was a waste of time. One participant from Malawi recorded in her voice diary, ‘I waited for a very long time to speak with an agent so it was really frustrating as I really needed help, but I just cut the phone since I couldn’t wait longer.’ Some women tried to multitask by putting calls on speaker to manage other responsibilities while waiting to be connected to an agent, but hotline agents noted this can distort audio and lead to dropped calls, adding to user frustration.

### Point of service

*Key Finding: Infrastructure and technical challenges as well as limited call center staffing exacerbated barriers related to women’s time constraints.*

Outdated technical infrastructure, poor network reception (especially in Mozambique), and limited staffing (especially in Malawi) exacerbated challenges users faced in reaching the hotlines. Key informants noted that dropped calls are common, and hotline agents reported being understaffed and unable to meet demand. Some agents felt pressure to shorten calls to accommodate high call volumes and expressed concern about implementing demand-generation activities without first addressing staffing gaps. These infrastructure and staffing issues led to frequent disconnections and unanswered calls, causing frustration among the women participants, many of whom were balancing other responsibilities. As a result, some women participants lost motivation to keep trying, with several describing the experience of reaching an agent as a matter of luck.

‘I called [AlôVida] four times. The first time I wasn’t answered. The second time I wasn’t answered. The third time I wasn’t answered. Then yesterday I tried to call. I think it was the problem with my phone. Sometimes the words didn’t reach [the agent] well. I don’t know if it was a problem with the network. So I couldn’t even speak… So today I didn’t try. I didn’t understand anything.’ – Ana, Mozambique.

*Key Finding: Free, 24/7 access to services in a variety of different languages drove the convenience of health hotlines, but this service goal was not always achieved, and poor service design exacerbated language barriers.*

Both countries’ hotlines are available 24/7 with multiple language options, and AlôVida is free for all users while CCPF is free only for AirTel subscribers. Key informants described efforts to staff shifts with both male and female agents who speak various languages, with higher staffing during the day. These factors contributed to the perceived convenience of services, especially in the evening or at night when clinics are closed. Women in Malawi generally found the language options satisfactory, as the IVR system allowed callers to select a preferred language before connecting with an agent, though some noted agents occasionally switched languages mid-call. In contrast, Mozambique’s IVR was only in Portuguese, with no option to choose a preferred language before reaching an agent. Most women were unaware that they could request to speak in a different language, leading to more frequent language mismatches and lower satisfaction. Poor service design in Mozambique, particularly around language access, limited some women’s ability to get the information they needed.

‘I think they could add other languages, or maybe ask in which language I would like to communicate with the agent.’ – Lúcia, Mozambique.

### Experience of care

*Key Finding: Women generally preferred speaking with agents if they could overcome the long wait times and disconnections. While the capacity of the IVR message platform is much higher, women had mixed reactions to the pre-recorded messages.*

Most women participants generally had positive experiences speaking with hotline agents, appreciating the clear explanations, attentive listening, and the convenience of not traveling to health facilities.

‘I was interested to learn how I can treat a child who has fever in the middle of the night and [the agent] was very helpful. He gave me good guidance and everything went well. It was just like speaking to a health worker at our health facility’ – Chifundo, Malawi.

However, satisfaction dropped when calls went unanswered or wait times were long. While IVR systems don’t face the same capacity constraints, most women preferred speaking with an agent, as they were used to having their health questions answered directly through in-person interactions with health workers or felt they could more easily get specific questions answered by agents rather than messages.

‘[I would prefer] speaking directly to the agent… I would get the response or feedback faster rather than waiting to receive a message or listening to recorded messages where I would listen to them and not have answers to questions I may have.’ – Eunice, Malawi.

Among those who listened to the messages, reactions were mixed. Some women found the messages informative, particularly those related to disease outbreaks. Others wished for more content on women’s health topics such as cervical cancer or pregnancy, especially in Mozambique where the current message topics are limited. Some women said the messages were too short and didn’t answer all their questions. Others said the messages were too long, didn’t fit into their schedules, or weren’t memorable, suggesting they may not have been engaging enough to hold attention. Recommendations included incorporating songs or dramas to make the messages both informative and entertaining.

### After service

*Key Finding: Call center data was not fully leveraged to make improvements to the services.*

Most key informants responded that only basic data is reviewed and used to inform staffing during different hotline shifts. However, there was limited review of gender data or of more detailed data such as call topics or outcomes, limiting the ability to tailor services to meet callers’ needs and preferences.

‘No, [I do not have an example of gender data being used to make a decision]. It is something we need to look at. We have been making this commentary that men call more for the service. But I think there was not yet a very strong concern in the sense of trying to solve this issue.’ - Hotline agent, Mozambique.

Additionally, there were no automated mechanisms in place to collect and assess user satisfaction with services, which further limited stakeholders’ insights into how services can be improved.

*Key Finding: Successful interactions with health hotlines led to more informed decision-making and behaviors around accessing other health services or implementing healthy behaviors.*

Many women participants noted that they felt more empowered with health information after their experiences with CCPF and AlôVida. In some cases, women learned how to treat health concerns like diarrhea at home. Others said they learned about hygiene and other healthy behaviors to prevent cholera.

‘I was also informed that during the rainy season in the time of diarrhea and colds that I could take care by covering the feces, washing the hands with soap. If there is someone with diarrhea they could take liquids, light coconut water, and hygiene above all.’ – Joana, Mozambique.

In some cases, participants received important information that helped them decide to seek further care at a facility.

‘I was encouraged [by listening to the messages] and I went to the hospital, and it was found [my daughter] had malaria… So, it is important, these messages have encouraged us not to take sickness lightly when a child says they are not feeling good.’ – Grace, Malawi.

## DISCUSSION

Despite their potential to provide timely, reliable health information, national health hotline services in Malawi and Mozambique remain underutilized by women. This study identified a variety of barriers limiting women’s uptake of services, including gender norms and geographical, socio-economic, cultural, and infrastructure factors. The findings largely align with the broader literature on digital health in LMICs, which highlight issues related to phone and network access, language and literacy challenges, low awareness of services, time burdens, and failure to design solutions for female users [3]. Findings also align with literature on health seeking behavior and decision-making norms that place caretaking responsibilities on women but added additional insights into how those norms impact how women tend to use digital health services and which kinds of services they prefer [20, 21]. The study also adds to the literature by emphasizing the importance of community-centered demand-generation strategies to reach women, such as through announcements at community meetings or markets, songs, facility health talks, activist teams, or women ambassadors.

The findings from this study differ from existing literature in several areas. While some previous studies have found privacy concerns to be a common barrier [22], our study found that perceptions of privacy and trust were mixed, with many women trusting the service due to its government affiliation but some still hesitant to discuss sensitive issues with people they didn’t know. This study found that most women felt that their family members would support them to use phones to access health information, contrary to previous literature on cultural norms restricting women from engaging with technology platforms [2, 8]. Finally, mobile phone access did not appear to be as significant a barrier for women in Malawi and Mozambique as is reported in the literature [4, 7]; even those participants who did not have their own phones largely reported being able to access phones from family or friends if they needed to call the hotline. This may reflect growing prevalence of and comfort with phones compared to when those studies were conducted. These discrepancies from the existing literature highlight the importance of designing context-specific solutions that are responsive to local cultural and social dynamics and that can adapt to a changing technology landscape.

### Limitations

This study has several limitations. Its qualitative design, sample size, and purposive sampling approach limit the generalizability of findings, especially those related to cultural and social norms. Additionally, insights into hotline structures and operations may be context-specific and primarily applicable to Malawi and Mozambique. The focus on women, while intentional, excluded perspectives from men and other key stakeholders, which may limit the breadth of findings. Additionally, some participants faced challenges using voice recorders, requiring extra orientation and repeated sessions, which may have affected response depth. Finally, the study reflects a specific timeframe and may not capture ongoing changes in access, technology, or policy in the dynamic digital health landscape.

### Study Implications

This study offers practical insights for improving national health hotline services in settings similar to Malawi and Mozambique, with a focus on better meeting women’s needs. By identifying structural, technical, and socio-cultural barriers, it highlights opportunities to address gender disparities in access to digital health. While some of the findings are specific to the design and structure of the CCPF and AlôVida hotlines, many of the lessons are also relevant for other digital health solutions in LMICs more broadly. The study highlights the need to consider language, digital literacy, gender roles, and caregiving responsibilities in service design and outreach strategies. It also identified key facilitators such as leveraging community structures and targeted communication, that may be relevant in other LMICs. For policymakers and implementers, the findings stress the need for robust technical systems, adequate human resources, data-driven decision-making, and sustainable financing to meet demand with reliable, high-quality services. It also points to the need for forward-looking strategies and funding models to enable infrastructure upgrades as technology advances. Further, the discrepancy between the hotlines’ official objectives to serve all citizens and some agents’ intentional focus on reaching men underscores the importance of aligning stakeholder perspectives with strategic goals. Such misalignment may contribute to lower female utilization if efforts to expand men’s engagement inadvertently deprioritize addressing barriers faced by women. In contexts where hotlines must triage calls due to limited capacity, stakeholders should be cautious that prioritization criteria do not inadvertently privilege men’s patterns of use. For instance, if calls concerning acute health issues are prioritized, women’s preventive and information-seeking calls may be deprioritized, exacerbating gender disparities in service access. Finally, the study highlights the importance of participatory research with target users to inform the design and ongoing improvements of digital health solutions.

## Supplementary Material

Supplemental_Material_Appendix_A_oqaf035

## Data Availability

Data cannot be shared for ethical/privacy reasons.
