# Supplementary material for: A qualitative, participatory study to identify barriers and facilitators to women’s uptake of National Health Hotline Solutions in Malawi and Mozambique
Source: Oxf Open Digit Health. 2026 Feb 6;4:oqaf035. doi: 10.1093/oodh/oqaf035 (PMC12888807; doi:10.1093/oodh/oqaf035)
Supplement: Supplemental_Material_Appendix_A_oqaf035 [file supplemental_material_appendix_a_oqaf035.docx]

**Supplemental Material Appendix A: Codebook**

| **Code Group** | **Code** | **Comment** |
| --- | --- | --- |
| Access to Assets | A.01 Access to own phone | Apply when participant mentions having her own phone |
|  | A.02 Access to shared phone | Apply when participant mentions having access to a shared phone (shared with another family member or a friend) |
|  | A.03 No/limited phone access | Apply when participant mentions not having phone access or having very limited access to a phone |
|  | A.04 Phone network | Apply when participant talks about level of availability of phone network / coverage |
|  | A.05 Phone charging | Apply when participants mention level of ability to charge phones. Any mention of power sources (solar, electricity, generators), batteries, charging cables, charging port, charing duration. |
|  | A.06 Awareness of hotlines | Apply when participants talk about women or general population being aware of hotlines and how they became aware. |
|  | A.07 Information sources | Apply when participants talk about sources of health information, for example: health workers, radio, friends/family, internet |
|  | A.08 Smartphone | Apply when participants describe using smartphones that have capabilities beyond just texts and calls |
|  | A.09 Basic phone | Apply when participants describe a feature phone or a traditional mobile phone. These are phones that focus on core functionalities such as voice calls and text messaging. |
|  | A.10 Services accessed | Apply when participant talks about health services (beyond just information) that she has accessed before |
|  | A.11 Service access challenges | Apply when participant talks about challenges when accessing health services (e.g. distance, money, etc.) |
|  | A.12 Service access facilitators | Apply when participant talks about facilitators of service access (e.g. transport to facility) |
|  | A.13 Information access challenges | Apply when participants describe difficulties and barriers to health information access (e.g. literacy, timing, language) |
|  | A.14 Information access facilitators | Apply when participants describe factors or interventions that improve access to health information |
|  | A.15 Financial resources | Apply when participants describe level of access to finances, financial resources related to health issues, strategies employed to cope with financial constraints, affordability of mobile phones, internet |
|  | A.16 Social networks and relationships | Apply when participants describe the role of family, friends, and community in providing support or hindrance, apply when participants describe how social networks and relationships contribute to/hinder accessing health resources |
|  | A.17 Digital literacy | Apply when digital literacy is mentioned / women's level of comfort with using phones or technology |
|  | A.18 Gendered differences in information access | Apply when there are gendered differences/preferences in accessing certain communication/promotional channels for example radios, TVs |
|  | A.19 Trust information/services | Apply when participants talk about trusting the health information or services they are using |
|  | A.20 Distrust in information/services | Apply when participants talk about not trusting the health information or services they are using |
| Beliefs and Perceptions | B.01 Importance of information | Apply when participant talks about how important or unimportant it is for her to have access to health information |
|  | B.02 Decision making processes | Apply when participants generally describe how decisions related to health are made within families |
|  | B.03 Male decisions | Apply when participant describes decisions that are usually made by men in her household |
|  | B.04 Female decisions | Apply when participant describes decisions that are usually made by women in her household |
|  | B.05 Joint decisions | Apply when participant describes decisions that are usually made jointly by men and women together in her household |
|  | B.06 Community norms on decisions | Apply when participant describes how other families in her community typically make decisions / if there is a difference between her family versus the rest of her community |
|  | B.07 Feelings about decision-making | Apply when participant describes how she feels about the types of decisions she can or cannot make |
|  | B.08 Gender norms / gendered expectations | Apply when participants mention gender norms, roles and expectations, when they mention how these expectations or norms influence their health seeking choices, decisions, behaviors |
| Practices and Participation | C.01 Division of responsibilities | Apply when participants describe generally how responsibilities related to health-seeking behavior are divided within the family |
|  | C.02 Female responsibilities | Apply when participants describe health-seeking responsibilities that are managed by women in her family |
|  | C.03 Male responsibilities | Apply when participants describe health-seeking responsibilities that are managed by men in her family |
|  | C.04 Joint responsibilities | Apply when participants describe health-seeking responsibilities that are jointly managed by men and women in her family |
|  | C.05 Community norms on responsibilities | Apply when participants describe how other families in the community generally divide responsibilities related to health-seeking behavior |
|  | C.06 Feelings about responsibilities | Apply when participants describe how they feel about how health-seeking responsibilities are divided within their households |
|  | C.07 Challenges managing responsibilities | Apply when participants describe challenges they experience carrying out their health-seeking responsibilities |
|  | C.08 Facilitators managing responsibilities | Apply when participants describe factors that improve their experiences when carrying out their health-seeking responsibilities, for example, family support, receptive providers, time management at clinics |
|  | C.09 Gender norms and responsibilities | Apply when participants describe how gender norms influence how responsibilities related to health care or caregiving are divided among family members |
|  | C.10 Time allocation and prioritization | Apply when participants describe how they allocate and prioritize time concerning health activities, and whether time constraints impact healthcare decisions. |
|  | C.11 Access to resources for managing responsibilities | Apply when participants describe level of access to resources for managing responsibilities (knowledge, money, transportation, time) |
|  | C.12 In-person interactions with health staff | Apply when participants talk about the types of in-person interactions they have with health staff or compare in-person interactions to remote/phone interactions |
|  | C.13 Female health behavior norms | Apply when participants talk about the normal health-seeking behaviors of women |
|  | C.14 Male health behavior norms | Apply when participants talk about the normal health-seeking behaviors of men |
| Institutions, Laws and Policies | D.01 Roles/responsibilities at hotlines | Apply when participant (agent or MoH staff) describes their roles or responsibilities related to running hotlines |
|  | D.02 Perceived goal of hotlines | Apply when participant talks about what they think the purpose or vision of hotlines is |
|  | D.03 Perceived target population of hotlines | Apply when participant mentions populations that hotlines should target |
|  | D.04 hotlines service uptake | Apply when participant talks generally about the level of uptake of hotlines services |
|  | D.05 Barriers to hotlines uptake | Apply when participant mentions obstacles to hotline uptake |
|  | D.06 Promotion / advertisement of hotlines | Apply when participant describes different channels (e.g. radio, TV, posters, etc.) for promoting hotlines and how the services are advertised (e.g. who is featured in advertisements, what the advertisements say, etc.) |
|  | D.07 Women inputs in hotlines design | Apply when participant talks about how women have been involved in the design of hotlines or provided inputs into the services |
|  | D.08 Capacity to handle gender issues | Apply when participant talks about whether hotlines is able to handle gender-related issues (e.g. are there enough women-specific health topics) |
|  | D.09 Male vs female agents / gender preferences | Apply when participant talks about the ratio of male vs female agents or user preferences on speaking with male vs female agents |
|  | D.10 Linkages outside of hotlines | Apply when participant talks about any linkages or connections that hotlines has with other departments (e.g. social services, police department, etc.) |
|  | D.11 Gender training/mentorship | Apply when participant talks about agents receiving training or mentorship on gender-related topics or how to communicate with female users |
|  | D.12 Hotline data / data-based decisions | Apply when participant talks about data and if/how data is used for decision-making related to hotlines services or other uses |
|  | D.13 Gender disparities in hotlines uptake | Apply when participant talks about differences in male uptake of hotlines vs female uptake including causes of disparities in uptake |
|  | D.14 Recommendations from agents/MoH | Apply when agents or MoH staff give recommendations on how to improve hotlines |
|  | D.15 Differences b/t male vs female callers | Apply when agents describe how they interact with male vs. female callers or any differences in how male vs. female callers use the hotlines |
|  | D.16 Men calling on behalf of women | Apply when agents talk about interactions with men who call on behalf of women users |
|  | D.17 Hotline languages | Apply when participants (agents or women) talk about the language options for hotline services or promotional activities |
|  | D.18 Hotline staffing/capacity | Apply when participants (agents or women) talk about the human resources capacity of hotlines / # of staff or level of burden of staff |
|  | D.19 Hotline feedback mechanisms | Apply when participants talk about existing or suggested mechanisms for collecting feedback from hotlines users |
|  | D.20 Skills / knowledge / training of hotline agents | Apply when participants talk about what kinds of skills and knowledge the hotlines agents have or don’t have or on what kind of training they've received on general or non-gender-specific health topics |
|  | D.21 Gender-specific data / data-based decisions | Apply when participants talk about gender-specific data or about making decisions based on gender-disaggregated data |
|  | D.22 Cross-sector engagement | Apply when participants talk about how hotlines can work with other branches of the MoH or other ministries / departments to improve services or further the goals of the hotlines |
|  | D.23 Hotline financial and technical resources | Apply when participants talk about availability or level of financial resources or technology resources impacting implementation or functioning of hotlines |
| Hotline / mHealth Experiences | E.01 Past experience with mHealth/hotlines | Apply when participants discuss past experiences accessing health information or services via phone |
|  | E.02 Motivation to use hotlines / perceived benefits | Apply when participants describe why they might choose to access health information or services via phone rather than in-person / or describe the perceived benefits of hotline services compared to other services. |
|  | E.04 Challenges to using hotlines | Apply when participants talk about challenges they experience when trying to use hotlines |
|  | E.05 Facilitators to using hotlines | Apply when participants talk about facilitators that make it easier for them to use hotlines |
|  | E.06 Trust in hotlines | Apply when participants talk about trusting hotlines / the information they get |
|  | E.07 Distrust in hotlines | Apply when participants talk about not trusting hotlines / the information they get |
|  | E.08 Satisfied with hotlines | Apply when participants talk about being satisfied with their experiences with hotlines |
|  | E.09 Dissatisfied with hotlines | Apply when participants talk about not being satisfied with their hotlines experiences |
|  | E.10 Other mHealth apart from hotlines (co-code) | Apply when participants talk about other phone-based health services apart from hotlines. Co-code on top of other codes. |
|  | E.11 Family members and mHealth utilization | Apply when participants talk about whether or not their family members use hotlines or other mobile health services |
|  | E.12 Attitudes of agents | Apply when participants talk about the attitudes of the hotline agents - e.g. were they respectful, were they listening to them properly, etc. |
|  | E.13 Interactions with agents | Apply as a general code when participants talk about any interactions they have with hotline agents |
|  | E.14 Experiences with IVR messages | Apply as a general code when participants talk about listening to IVR messages |
|  | E.15 Other desired services/topics | Apply when participants talk about other services or health topics they would like to be able to access through hotlines / mobile health services |
|  | E.16 Recommendations from users | Apply when women participants give recommendations or feedback for how hotlines could be improved |
|  | E.17 Preferred service channels/platforms | Apply when participants talk about which service channels (e.g. IVR, hotline, USSD, Whatsapp, etc.) they prefer to use |
|  | E.18 Comfort with hotlines / privacy concerns | Apply when participants describe how comfortable they are using hotlines - e.g. do they have privacy concerns or not. |
|  | E.19 Family attitudes towards hotlines | Apply when participants describe their family members' reactions to them using hotlines or how their family feels about mobile health services |
|  | E.20 Call not answered / long wait time | Apply when participants talk about calls not being answered / long wait times when calling the hotline |
|  | E.21 Navigation through menu | Apply when participants talk about their experiences navigating through the IVR menu - e.g. to select to speak with an operator or to navigate to the IVR messages they want to listen to |
|  | E.22 Quality of communication / language | Apply when participants talk about the level of quality of the communication and language when they speak with an operator - can they hear the operator clearly, can they understand the operator, etc. |
| Health Topics | T.01 Immunization / child health | Apply when participant talks about immunization or child health topics |
|  | T.02 Family planning | Apply when participant talks about family planning topics |
|  | T.03 SRH | Apply when participant talks about SRH topics |
|  | T.04 Maternal health | Apply when participant talks about maternal health topics |
|  | T.05 General health | Apply when participant talks about general health topics |
|  | T.06 HIV/AIDS | Apply when participant talks about HIV/AIDS topics |
|  | T.07 Adolescent health | Apply when participant talks about adolescent health topics |
|  | T.08 Disease-specific / outbreak topics | Apply when participants refer to hotlines service providing information around specific diseases, e.g. outbreaks |
|  | T.09 Cervical cancer | Apply when participant talks about cervical cancer topics |
|  | T.10 Nutrition | Apply when participant talks about nutrition topics |
|  | T.11 Mental health | Apply when participant talks about mental health topics |
|  | T.12 COVID-19 | Apply when participant talks about COVID-19 topics |
| Other | ZZ. Come back and review | Apply for passages that you want to revisit or discuss with the group |
|  | ZZ. Quotable | Apply for quotes that are really powerful or convey a theme really well |
